# Optimization of High-Throughput Multiplexed Phenotyping of Extracellular Vesicles Performed in 96-Well Microtiter Plates

**DOI:** 10.3390/polym13142368

**Published:** 2021-07-19

**Authors:** Malene Møller Jørgensen, Jenni Kathrine Sloth, Rikke Bæk

**Affiliations:** 1 Department of Clinical Immunology, Aalborg University Hospital, 9000 Aalborg, Denmark; jennisloth@gmail.com (J.K.S.); rikke.baek@rn.dk (R.B.); 2 Department of Clinical Medicine, Aalborg University, 9000 Aalborg, Denmark

**Keywords:** extracellular vesicles, microarray, EV array, phenotyping, storage conditions, incubation buffers, spotting buffers

## Abstract

Extracellular vesicles (EVs) are promising biomarkers for several diseases, however, no simple and robust methods exist to characterize EVs in a clinical setting. The EV Array analysis is based on a protein microarray platform, where antibodies are printed onto a solid surface that enables the capture of small EVs (sEVs) by their surface or surface-associated proteins. The EV Array analysis was transferred to an easily handled microtiter plate (MTP) format and a range of optimization experiments were performed within this study. The optimization was performed in a comprehensive analytical setup where the focus was on the selection of additives added to spotting-, blocking-, and incubation buffers as well as the storage of printed antibody arrays under different temperatures from one day to 12 weeks. After ending the analysis, the stability of the fluorescent signal was investigated at different storage conditions for up to eight weeks. The various parameters and conditions tested within this study were shown to have a high influence on each other. The reactivity of the spots was found to be preserved for up to 12 weeks when stored at room temperature and using blocking procedure IV in combination with trehalose in the spotting buffer. Similar preservation could be obtained using glycerol or sciSPOT D1 in the spotting buffers, but only if stored at 4 °C after blocking procedure I. Conclusively, it was found that immediate scanning of the MTPs after analysis was not critical if stored dried, in the dark, and at room temperature. The findings in this study highlight the necessity of performing optimization experiments when transferring an established analysis to a new technological platform.

## 1. Introduction

Novel extracellular vesicle (EV) based diagnostic techniques are promising non-invasive procedures for early stage disease detection that are gaining increasing importance in the medical field. EVs are cell derived particles found in body liquids, particularly in high numbers in blood, from which they are isolated for further analysis [1]. EVs are involved in cell-to-cell communication, they carry biological material from the cell of origin, and they are transported throughout the human body by all body fluids [2,3]. Many studies have suggested that EVs are promising biomarkers for diseases such as metastasis of cancer, diabetes, coronary diseases, inflammatory diseases, infectious diseases, neurologic diseases, and others as reviewed by Schou et al. [4]. Furthermore, EVs can be collected rather non-invasively and give information about the cells from which they originate.

Although the EV phenotype is particularly important in the determination of cellular and subcellular origin, it can, in combination with a protein cargo analysis, additionally provide clues about the functionality of the EVs. Further refinements of existing methods will not only contribute to broadening our understanding of the biological role of the EVs, but are also likely to accelerate the implementation of EVs as biomarkers in clinical diagnostics [5,6]. Several methods exist to characterize the protein composition of EVs related to either a surface marker phenotype or the proteins present in the EV cargo, as reviewed by Revenfeld et al. [7] and Panagopoulou et al. [8]. Detection and molecular profiling of EVs is technically challenging and often requires extensive sample purification and labelling [9,10,11]. 

Previously, we developed and described a high-throughput approach for phenotyping EVs [12,13]. This approach, termed the ‘‘EV Array’’, is based on a protein microarray platform. Antibodies are printed onto epoxy-coated glass slides, which enable the capture of small EVs (sEV) by their surface or surface-associated proteins. Afterwards, profiling of the EVs is performed by detection with selected biotinylated antibodies for sEVs, e.g., anti-CD9, -CD63, and -CD81. 

The EV Array analysis requires manual handling of the glass slides throughout all steps. Handling slides requires a fast and trained workflow to avoid inappropriate drying of the surface and great precautions not to touch the spotted microarray. To run the analysis in a high-throughput manner, the slides were assembled in a multi-well gasket, where a silicone membrane was divided into 96 wells. A perfect assembling is crucial to avoid leakages between the well. 

To bring the EV Array technology into a clinical setting in the future will require development of a more robust and easily handled analysis, which is why a replacement for the slides as microarray substrates is needed.

Variants and derivatives of immunological analyses in 96-well microtiter plates (MTP) have become assay workhorses of laboratories, and a host of compatible reagents, consumables, plate washers, multi-channel pipettes, robotic liquid handlers, and assay formats have been developed and are available from multiple vendors. An alternative format suitable for use in research labs is a MTP-based microarray printed directly onto the bottom of a 96-well plate [14]. This method has been used by different investigators and companies. For example, SearchLight™ arrays (Pierce Biotechnology, Rockford, IL, USA) have up to 16 assays per well in a sandwich format similar to other multiplexed sandwich microELISA arrays [15]. Likewise, our EV Array analysis was transferred to such a MTP format and a range of optimization experiments were performed.

To capture EVs onto a surface, the maintenance of the capture bioactivity and performance of antibodies on microarray substrates for relatively long periods (under storage or shipping) requires carefully designed and optimized conditions. Here, we focused on the selection of additives added to spotting-, blocking-, and incubation buffers and the storage of printed antibody arrays under different temperatures from one day to 12 weeks. After the analysis ended, the stability of the fluorescent signal was investigated at different storage conditions for up to eight weeks.

## 2. Materials and Methods 

### 2.1. Production of the EV Array

For this test, 18 MTPs of the type Microfluor 2 (96-wells, polystyrene, round well, flat bottom, black, Thermo Fisher Scientific, Waltham, MA, USA) were prepared using a sciFLEXARRAYER S12 microarray printer installed with a piezo dispense capillary (PDC) size 60 with coating type 3 (Scienion AG, Berlin, Germany. The printing procedure was performed under strict humidity (55–65%) and temperature control (18–20 °C).

**Figure 1:**
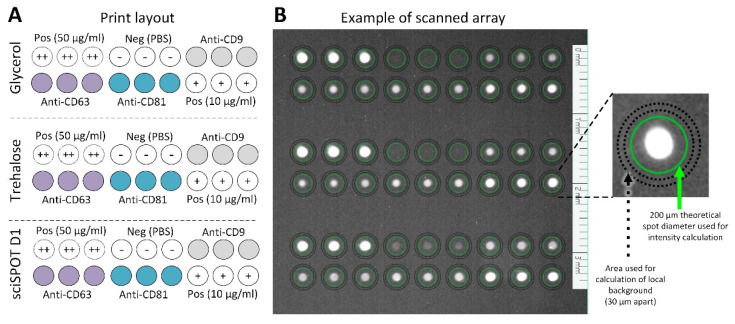
(**A**) Sketch of the microarray printed in each MTP well. A sub-array was created for each of the spotting buffers (containing glycerol, trehalose, or sciSPOT D1) and each analyte was printed in triplicate. (**B**) Example of one MTP well fluorescently scanned after the EV Array analysis. The magnified spot illustrates the template for the calculation of the mean spot intensity and local background subtraction.

The design of the array (shown in Figure 1) consisted of three anti-human antibodies against CD63 (Bio-rad Laboratories Inc., Hercules, CA, USA), CD9, and CD81 (Ancell Corporation, Stillwater, MN, USA) at a concentration of 100 µg/mL, biotinylated goat anti-mouse IgG (Novus Biologicals, Centennial, CO, USA) in two concentrations (10 µg/mL and 50 µg/mL) as positive controls, and buffer as the negative control. The antibodies and controls were prepared in three different spotting buffers: 5% glycerol in PBS (denoted “gly”), 50 mM trehalose in PBS (denoted “tre”), and 1 × sciSPOT Protein D1 buffer (2 × concentrate, denoted “D1”) in PBS (Scienion AG, Berlin, Germany), which were all included in the same print design. When the printing was finalized, the MTPs were incubated in the controlled climate of the printer for 10 h to allow adsorption of the antibodies.

### 2.2. Blocking and Storage

All blocking procedures were performed at room temperature (RT). Three of the blocking procedures were initiated prior to storage. Each MTP was divided into four areas, one for each blocking procedure. The blocking procedures were performed one by one while the other three areas were sealed off. By the end of the pre-storage blocking, the plates were sealed with adhesive film.

For blocking I, blocking buffer (50 mM Ethanolamine, 0.1% SDS, 100 mM Tris, pH 9.0) was applied using a hand-held spray gun in a closed box for gentle application of the buffer. After 30 min of incubation, an additional 100 µL of blocking buffer was added to each well, followed by another 30 min incubation. Subsequently, the wells were emptied and left to dry for 5 h.

Blocking II was performed similar to blocking I, but after the storage period. This procedure, however, was finalized after the first 30 min of incubation.

For blocking III; 100 µL of Liquid Plate Sealer^®^ (Candor Bioscience GmbH, Wangen im Allgäu, Germany) was gently added to each well with a multi-channel pipette. After 1 h of incubation, the liquid was removed, and the wells were left to dry for 5 h.

Blocking procedure IV is a combination of blocking I and III. Initially, blocking procedure I was performed. After 30 min drying time, blocking procedure III was performed.

Two MTPs were prepared for analysis immediately after the blocking procedures by performing blocking II alongside the other blocking procedures. One MTP was analyzed directly after incubation in the box, whereas the other MTPs were left to dry for 2 h before analysis. Of the remaining MTPs, half of them were placed at RT and the other half were stored at 4 °C. One MTP from each storage condition was analyzed after 1, 2, 3, 4, 6, 8, 10, and 12 weeks of storage, respectively.

### 2.3. Sample Preparation

All research involving samples from human subjects was approved by the local ethics legislation. Each person signed a written consent form allowing for the use of their blood for research purposes. Venous peripheral blood was obtained from three healthy individuals at Aalborg University Hospital (North Region, Aalborg, Denmark) using CPDA tubes (Vacuette^®^, Greiner Bio-One, Kremsmünster, Austria). Plasma was collected after centrifugation at 1.800× *g* for 6 min at RT and mixed to homogenize, aliquoted, and stored at −40 °C until further analysis.

For the analysis, two different incubation buffers were prepared: Buffer A (½ × Casein Blocking Buffer (10 × concentrate, Sigma-Aldrich, St. Louis, MO, USA, catalog B6429) and 0.1% Tween20^®^ in PBS), and Buffer B (0.2% Tween20^®^ in PBS). A large portion of the three different plasma samples in four dilutions were prepared in the two buffers resulting in final sample-analysis-volumes of 0, 25, 50, and 75 µL in a total volume of 100 µL. The diluted samples were aliquoted in nine portions and stored at −40 °C, hence, for each analysis of a set of two MTPs, a freshly thawed portion of the same sample stocks could be used to eliminate pipetting variations.

### 2.4. Analysis and Scanning

The EV Array analysis was initiated by washing the MTPs in Buffer B using a HydroFlex™ microplate washer (Tecan Trading AG, Männedorf, Switzerland). Then, 100 µL of the sample was applied to each well and incubated for 2 h RT in an orbital shaker (450 rpm) followed by an overnight incubation at 4 °C. After a wash procedure in Buffer B, each well of the MTPs were incubated with 100 µL detection antibody cocktail (biotinylated anti-human-CD9, -CD63, and -CD81 (Ancell Corporation, Stillwater, MN, USA) diluted 1:1500 in Buffer A and B, respectively) for 2 h RT with shaking. Following a wash in Buffer B, 100 µL of streptavidin-Cy3 ((Life Technologies, Carlsbad, CA, USA) diluted 1:3000 in each buffer) was added to each well and incubated for 30 min RT on the shaker. The analysis was finalized by washing with Buffer B and subsequently with MilliQ water.

The MTPs were dried and scanned using a sciREADER FL2 microarray scanner (Scienion AG, Berlin, Germany) at 535 nm and an exposure time at 2000 ms.

The first two MTPs were saved for rescan after 1, 2, 3, 4, 6, and 8 weeks. One MTP was sealed and stored dry at RT, whereas 100 µL MilliQ water was added to each well of the other MTP before sealing and storage at 4 °C. Both MTPs were kept in the dark during the storage time.

### 2.5. Data Analysis

The sciREADER FL2 software (Scienion AG, Berlin, Germany) was used to obtain the mean spot intensities from a fixed spot size at Ø200 µm and calculated in relation to the local spot background positioned 30 µm from the outer diameter of the spot (Figure 1, magnified insert).

Calculations, graphs, and heatmaps were created using either Microsoft Excel 365 (Redmond, WA, USA) or GraphPad Prism 8 (GraphPad Software, LLC, San Diego, CA, USA).

## 3. Results and Discussion

We used the multiplexed, highly sensitive, and high throughput platform of the EV Array as a basis for optimizing the method to be performed in microtiter plates. To assure detection of the broadest possible EV collection, it was decided to use detection antibodies against human CD9, CD63, and CD81, concurrently. All three antigens were targeted using a cocktail of the antibodies to maximize the detection signals. These antibodies were chosen because they are known to be present on sEVs.

The ability for the EV Array to capture sEVs in a quantitative manner have previously been proven within other works, where detailed molecular analyses of the sEVs were performed by nanotracking analysis (NTA) [12], transmission electron microscopy (TEM) [16,17], and western blotting [18]. The scope of this study was to optimize an already established and verified technology, which is why we chose not to focus on the EV characteristics despite the recommendations by the MISEV guidelines [9].

Prior to this study, a pilot study was performed, and the tested materials are described in Supplementary Materials S1. The pilot study included 13 variants of MTPs, six variants of spotting buffers, eight variants of blocking buffers, and five variants of wash and incubation buffers. The results (not shown) from the pilot study were used to confirm which parameters should be included in this comprehensive optimization study.

### 3.1. Experimental Setup

**Figure 2:**
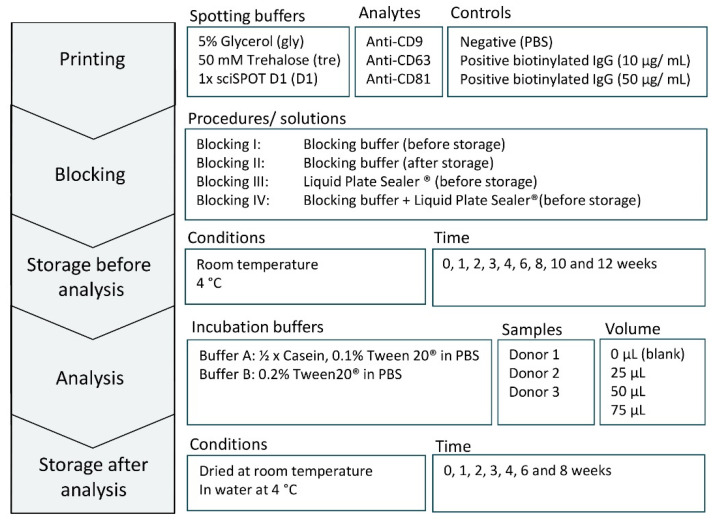
Sketch of the experimental setup. In the printing procedure, all analytes and controls were printed in each of the three spotting buffers. Four procedures were tested for the blocking step prior to storage at either RT or 4 °C before analysis, either immediately after the printing procedure (day 0) or after up to 12 weeks of storage. Samples from three donors were analyzed in incubation Buffers A or B with four different sample volumes. The MTPs were scanned after ending the analysis and the stability of the fluorescence was afterward tested at two conditions for up to eight weeks.

For the EV Array analysis, microtiter plates (MTP) were used as the basis of the array where a design of antibody spots was placed in the bottom of each well. The different parameters tested throughout the whole analysis is outlined in Figure 2.

For each time point, two plates were analyzed corresponding to more than 10,000 spots (2 plates × 96 well × 54 spots), so after all nine time points, a total of more than 90,000 spots/data points were analyzed. To control the stability of the fluorescence after analysis, all spots of the two first MTPs were re-analyzed at six time points. Hence, in total, more than 155,000 of data points were included in this study.

**Figure 3:**
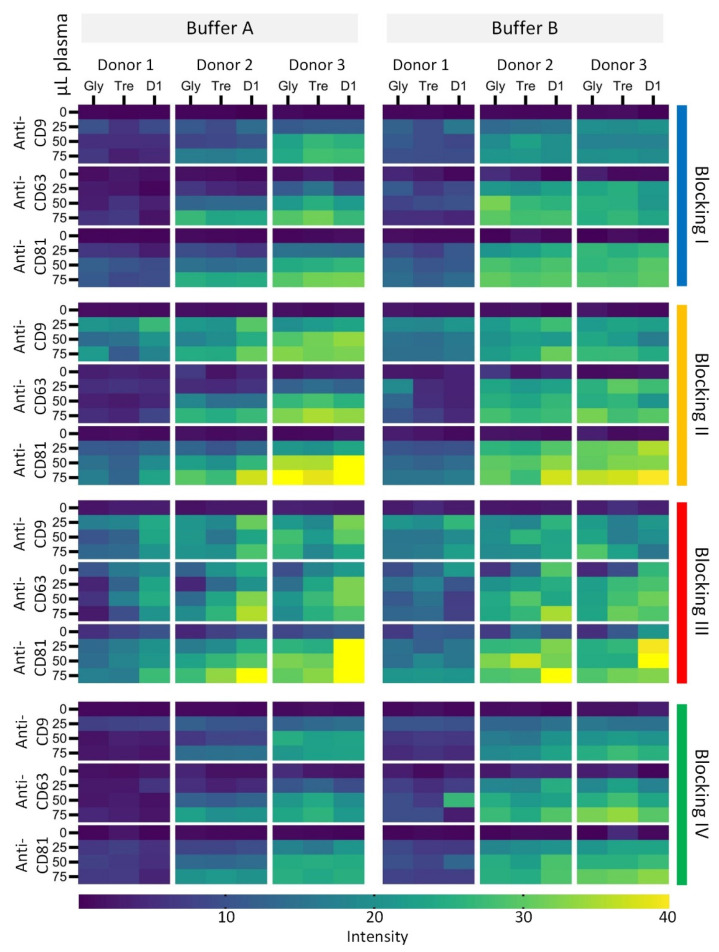
Heatmap summarizing the spot intensities (signal-to-background ratios) from anti-CD9, anti-CD63, and anti-CD81 spots obtained from one MTP blocked and analyzed without storage.

A summary of the data collected from the anti-CD9, anti-CD63, and anti-CD81 spots from one MTP at the starting point is illustrated in Figure 3. The spot intensities (background corrected) can be seen to vary greatly. Biological variation between the donors were, as expected, observed in the heatmap, with donor 1 having the lowest signal intensities.

**Figure 4:**
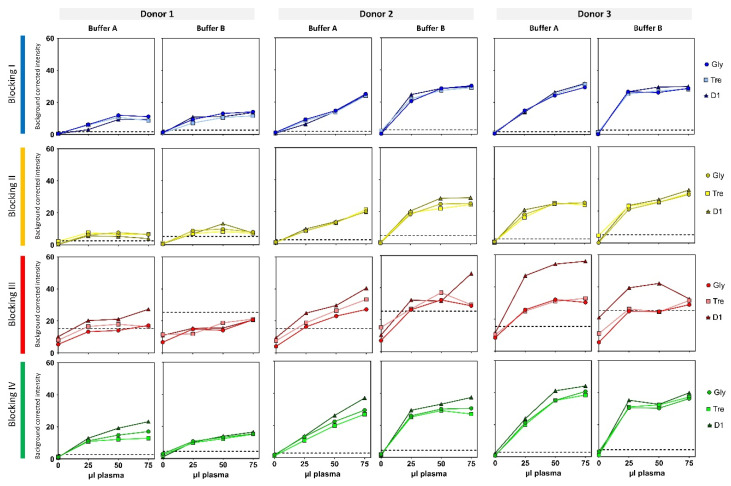
Background corrected intensities of spots containing anti-CD81 for printing buffer containing glycerol, trehalose, or sciSPOT D1 for four blocking procedures, both incubation buffers, and all three donors plotted against plasma volume added. Dotted lines indicate limit-of-detection calculated as 3SD of the sample without plasma (0 µL).

The change of analysis format from epoxy-coated glass slides to plastic MTPs required new equipment for the scanning procedure. Going from a high-resolution (16 bit and down to a pixel size of 3 µm) laser-based confocal scanning (Innoscan 710 AL, Innopsys, Carbonne, France) to a LED-based image acquisition (8-bit and a pixel size of 7 µm) decreased the sensitivity of the EV Array significantly. The original EV Array analysis was performed using a sample volume of 10 µL plasma, whereas the new optimal sample volume was tested within this experimental setup (Figure 3 and Figure 4). In order to obtain the best signal-to-noise ratio, it was found that 75 µL of plasma was required to be able to analyze samples with small amounts of vesicles (donor 1), even though it comprises that signals are saturated in donors with high amounts of vesicles (Figure 4, donor 2 and 3).

### 3.2. Spotting Buffer

It is of great importance to efficiently immobilize probes onto a substrate with good spot quality for the fabrication of protein microarrays. The surface properties of substrates affect the spot quality of protein microarrays, and an appropriate spotting buffer can significantly improve the surface binding capacity, the stability of proteins over time and the quality of the spots produced. Unlike DNA, proteins (antigens and antibodies) have 3-dimentional structures and easily lose their reactivities due to dehydration and denaturation during array printing and immobilization. The ability to protect the antibodies against denaturation is one of the most challenging tasks in the fabrication of protein microarrays. Even at RT and in humid environments, the nanodroplets containing proteins spotted on the surfaces could quickly evaporate to cause protein dehydration and denaturation. Several studies have investigated the effect of pH in spotting buffers on the protein immobilization [19] and application supplements such as glycerol [20,21], trehalose [19,22], saccharose [23], sciSPOT buffers [24], or other additives [25] to prevent dehydration and denaturation of immobilized proteins. Even though these supplements can prevent dehydration and improve the signal intensity, the non-uniform spot structure can still occur, which will result in poor reproducibility and difficulty in quantitative application of protein microarrays [26].

Prior to this study, a pilot study was performed (data not shown) to test various spotting buffers and their properties. The tested buffers were 5% glycerol in PBS, 50 mM trehalose in PBS, 1 × sciSPOT D1 in PBS, 1 × sciSPOT D4 in PBS, 1 × sciSPOT D11 in PBS (Scienion AG, Berlin, Germany), and 50 mM carbonate (pH 9.6). Of these, only 5% glycerol, 50 mM trehalose, and 1 × sciSPOT D1 were included in this experimental setup. The remaining buffers were found to be not compatible with the plastics, coatings, or was highly affected by static electricity in the printing process, causing the drops to dislocate.

**Figure 5:**
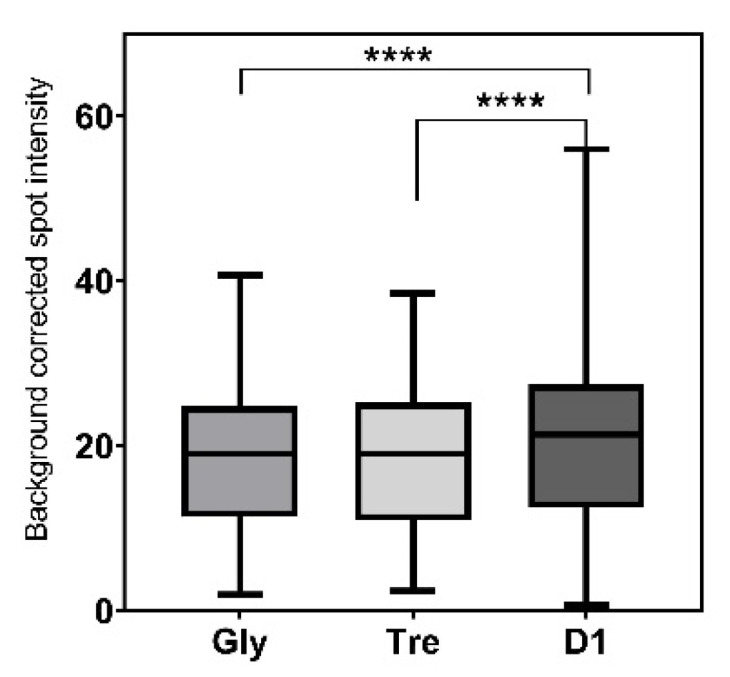
Box and whiskers plot (min to max) of the background corrected intensities for the spotting buffers. Data include spots containing anti-CD9, anti-CD63, and anti-CD81, four blocking procedures, and three donors. ****; *p* > 0.0001 as determined using a paired Wilcoxon test.

Hence, the effect of adding glycerol, trehalose, or sciSPOT D1 to the spotting buffer to prevent dehydration and keep the stability of the printed antibodies during storage was investigated. The abilities of the antibodies to capture sEVs in the various spotting buffers prior to storage are seen in Figure 3, Figure 4 and Figure 5.

The best absolute signal intensities and signal-to-background ratios were obtained using sciSPOT D1. Spots printed with sciSPOT D1 were found to be significantly higher (*p* < 0.0001) than both glycerol and trehalose using a paired Wilcoxon test. When using blocking IV, the spots containing trehalose were found to gain the best signal intensities (Figure 3). This exemplifies how the various tested parameters and conditions within this study had a high influence on each other. This underlines why a systematic test of all the combinations is needed to find the most optimal conditions to capture and analyze sEVs.

The interactions between the spotting buffers and blocking buffers were seen to be affected even more after storage prior to analysis, which is described in the next sections.

### 3.3. Blocking and Storage Prior to Analysis

Blocking is essential in any antibody/antigen relationship. The correct blocking buffer can perfect the antibody′s ability to bind to its antigen, while bad blocking can make specific antibody binding near impossible. Unspecific background signal (antigen binding in the absence of antibody) is one of the most serious, if not most severe, problems encountered in the protein microarray technology. No blocking reagent or method is ideal for all assays. The Microfluor 2 plates used in this study are made of slightly hydrophilic polystyrene and passively binds a diverse range of biomolecules. Blocking agents are typically ethanolamine for protein coupling via amino groups in addition to a low content of an ionic detergent (SDS) [27]. Further reduction of unspecific binding can be obtained by the addition of proteins to the blocking buffer; however, this was found not to be the case when testing it in the pilot study.

Four blocking procedures were tested in this study; blockings I, II, and IV were performed with a slight alkaline ethanolamine containing buffer with low amounts of SDS. Blocking I was performed prior to storage and blocking II after storage. The commercially available Liquid Plate Sealer^®^ was used as the blocking reagent in blocking III and this reagent was also added in blocking IV prior to storage. The Liquid Plate Sealer^®^ was developed as a stabilizer for antibodies and antigens for longer periods of storage [28]. The content of the Liquid Plate Sealer^®^ is unknown.

Analysis performed without storage (day 1) showed higher signals when using blockings II and III relative to blockings I and IV (Figure 3 and Figure 4). However, signals were also found in blanc/negative wells (0 µL plasma) in spots containing anti-CD63 and anti-CD81 (Figure 3) when using blockings II (minor signals) and III (major signals), raising the limit-of-detection (3SD–dotted lines in Figure 4). This indicates that the unspecific bindings to these antibodies are dependent of the blocking procedure. Therefore, if the analysis is to be performed on freshly prepared arrays, blocking procedures I and IV are recommended in combination with the spotting buffers containing sciSPOT D1 and trehalose, respectively.

The ability to store microarrays prior to analysis is an important time- and material-saving element in microarray technology, since it allows bulk production. Previous studies of Diego-Castilla et al. [29] revealed that antibody arrays are stored best at 4 °C or RT in comparison with −80 °C or 37 °C. Therefore, after blocking and drying, the MTPs were sealed with tape and stored either at RT or at 4 °C for up to 12 weeks.

**Figure 6:**
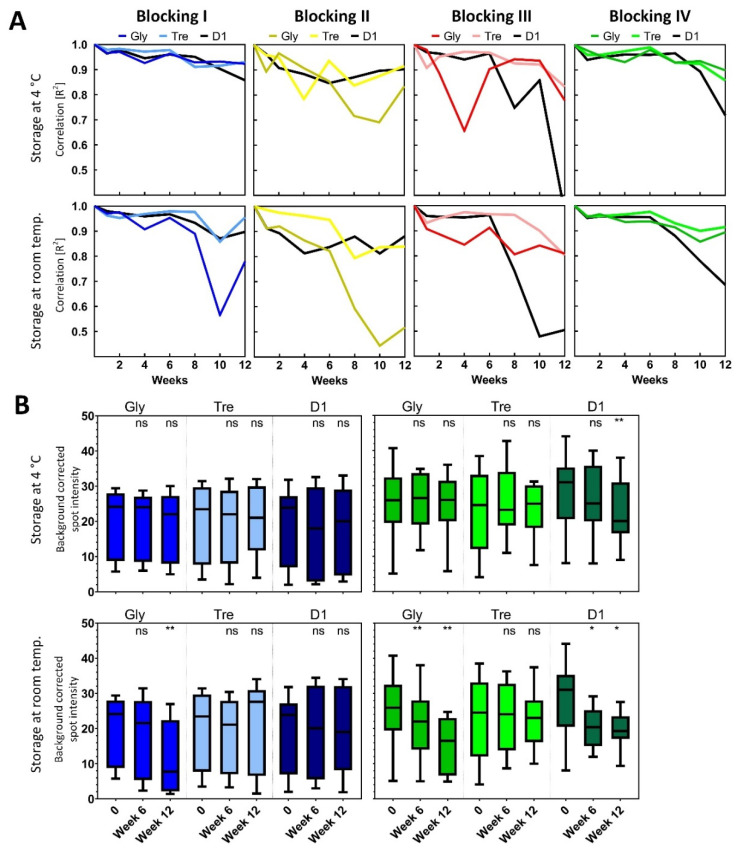
(**A**) Coefficient of determination (R^2^) was obtained when plotting all spot intensities (across both incubation buffers) against the corresponding intensities at day 1. The coefficients (correlations) are depicted for each blocking buffer, storage condition for storage prior to analysis, and spotting buffer used. (**B**) Box and whiskers plot (min to max) of the background corrected intensities obtained from anti-CD9, anti-CD63, anti-CD81 spots prior to storage and after six and 12 weeks of storage. Left/blue panel; blocking procedure I; Right/green panel; blocking procedure IV. * *p* < 0.05; ** *p* < 0.01; ns not significant.

The EV analysis was repeated after storage for 1, 2, 3, 4, 6, 8, 10, and 12 weeks. Spot intensities for anti-CD9, anti-CD63, anti-CD81, and the positive controls (biotinylated IgG) were plotted against the spot intensities from the EV analysis performed at day 1 and the correlation (R^2^) was calculated. In Figure 6A, the correlations are depicted for each blocking procedure, storage condition, and spotting buffer used.

The effect of the storage is seen as a decrease in the coefficients of determinations (R^2^) over time. Generally, it is seen that storage at RT gives a decrease in the correlations earlier than with storage at 4 °C. Blocking procedures II and III are not good options for preserving the reactivity of the spotted antibodies over time, independent of the storage conditions and spotting buffer. Blocking II was performed immediately prior to the analysis, so the preservation of the antibody reactivity was solely due to the effect of the spotting buffer.

Using blocking procedure I, no significant reduction of reactivity was seen after 12 weeks of storage at 4 °C, however, when stored at RT, a significant reduction (*p* < 0.01) was seen when using glycerol containing spotting buffer (Figure 6B). Compared to six weeks of storage, a 62% reduction in mean signals was observed after 12 weeks.

sciSPOT D1 containing spotting buffer showed a significant reduction of reactivity after 12 weeks of storage at 4 °C when using blocking procedure IV. This reduction was not observed when using glycerol or trehalose. However, a significant reduction in reactivity was already seen after six weeks of storage at RT when using glycerol (*p* < 0.01) and sciSPOT D1 (*p* < 0.05).

The best preservation of the spots (independent of storage temperature) was found when using trehalose in the spotting buffer and in combination with either blocking procedures I or IV (Figure 6B). Similar preservation could be obtained using glycerol or sciSPOT D1 in the spotting buffers, but only if stored at 4 °C after blocking procedure I.

Using the Liquid Plate Sealer^®^ (blocking III) showed not to prolong the storage time of the spotted array, however, it tended to prolong the storage time at RT when used in combination with ethanolamine (blocking IV) and spotting buffer containing trehalose.

Conclusively, it was found that the antibody arrays can be stored up to 12 weeks, even at RT without affecting the reactivity when using blocking procedures I or IV and trehalose in the spotting buffer, and also possibly for longer as a %CV of 25 is fully acceptable for immunoassays [30]. 

### 3.4. Incubation Buffers

To capture and detect EVs in biological samples, it is important to keep the vesicles intact. To avoid interruption of the vesicles due to osmotic stress, PBS was used as the basic buffer in the incubation and washing procedures of the EV Array analysis. Working with immunoassays and complex samples as serum or plasma non-specific binding interactions must be minimized, and for this purpose, the non-ionic detergent Tween20^®^ was conventionally included in wash and incubation buffers [31,32,33]. However, Tween20^®^ alone has not been proven to be an adequate blocking agent in all instances, and where this is the case, the addition of a non-reactive protein (commonly BSA or casein) to these buffers has sometimes, but not always, proved to be of value [32].

Within the pilot study, it was found that the addition of casein had an overall positive effect on the performance of the EV Array, reducing the unspecific bindings. Therefore, incubation buffers with (Buffer A) and without (Buffer B) casein were tested in this study. The results are presented in Figure 3 and Figure 4, where it is seen that the presence of casein generally tends to decrease the spot intensities, which indicates a reduction in unspecific bindings. Looking in more detail in Figure 3 and Figure 4 for blocking procedures I and IV, it can be seen that an increased sample volume also resulted in increased spot intensities and not showing a saturation of the signals when using incubation Buffer A. This was not seen when using incubation Buffer B.

**Figure 7:**
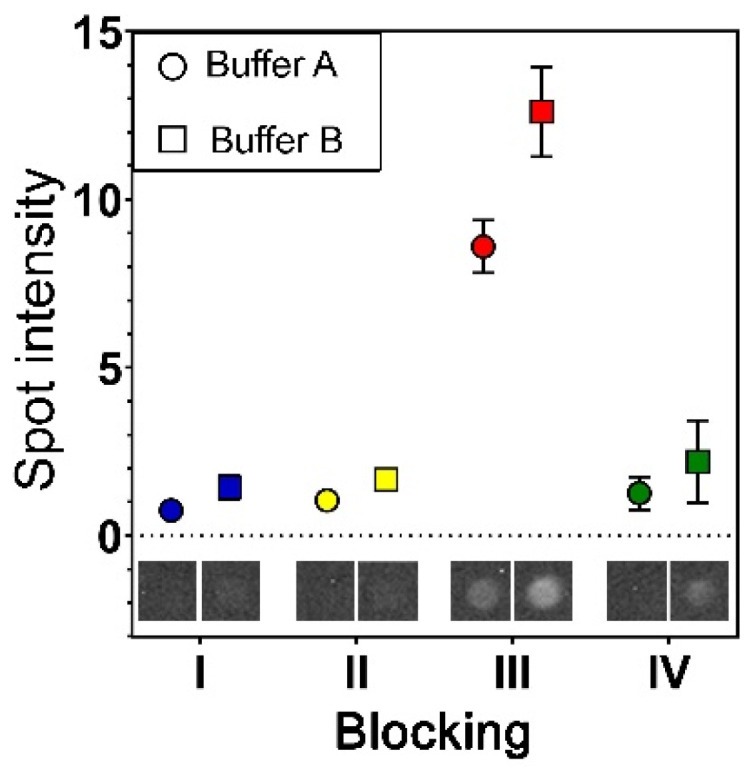
Unspecific binding between the printed antibodies and detection antibodies were found to be dependent on the incubation buffer and blocking procedure used. This is exemplified by spot images and data obtained from the anti-CD81 Spot.

Additionally, a higher level of unspecific binding was found between the spotted antibodies and the detection antibodies (no sample present) when using incubation Buffer B (Figure 7). These unspecific bindings were found to be significant when using blocking procedure III, raising the limit-of-detection (Figure 4).

### 3.5. Storage Post-Analysis

In general, fluorescent dyes are sensitive to light exposure as well as other environmental factors such as water, high temperature, alkalic pH, and alcohol. Evidence indicates that even ozone levels in the laboratory atmosphere could affect fluorescent dyes on microarrays [34]. In order to avoid or minimize the effects of these risk factors, it is appropriate to scan microarray slides instantly upon finishing the final wash and drying processes without unnecessary delays. Gu et al. [35] tested the signal stability of antigen-antibody arrays labelled with Cy3 and Cy5 and found only a slight decrease with storage at −20 °C for up to 30 days. However, when analyzing EVs, it would be questionable whether the freezing procedure would burst the vesicles. Therefore, this study additionally included testing the storing limits of the analyzed MTPs either dried at RT or containing water at 4 °C.

**Figure 8:**
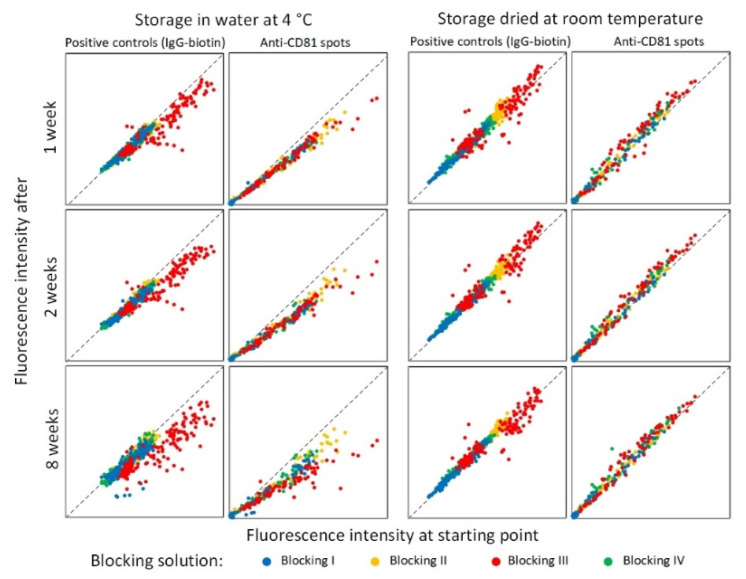
Scatter plots showing the stability of the fluorescence signal after ending the analysis. Spot intensities for anti-CD81 and positive control (IgG) spots across the two incubation buffers are depicted. MTPs were stored either containing water at 4 °C or dried at RT. Colors indicate the blocking procedures.

To determine the stability of the fluorescence from Cy3 after analysis, all spots of the two first MTPs were re-scanned and analyzed at six time points (1, 2, 3, 4, 6, and 8 weeks). The results from weeks 1, 2, and 8 are shown as a scatter plot in Figure 8. The data presented are from the positive control spots (Biotinylated IgG) and from the anti-CD81 spot capturing the sEVs.

Already after one week of storage in water at 4 °C, the signals were fading, and it continued to fade throughout all eight weeks. This was seen for both the positive control spots and the anti-CD81 spots, indicating that fading is caused by fluorescent signal destabilization and not the rupture of the sEVs. In contrast, no decrease in fluorescent signal was seen when the MTPs were stored dried at RT. These results show that with the presented analytical combinations of surface, spotting buffers, blocking procedures, and incubation buffers, the fluorescent signals from Cy3 are highly stable and immediately scanning is not critical. This opens up the possibilities of using an external scanning service if needed.

## 4. Conclusions

The change of the EV Array analysis format from epoxy-coated glass slides to plastic 96-well MTPs required the optimization of the procedure and components used throughout the analysis.

The slightly hydrophilic Microfluor 2 polystyrene plates were used as the template and the microarray was printed directly onto the bottom of each well. Spotting buffers containing glycerol, trehalose, or sciSPOT D1 were used in combination with positive controls (IgG), anti-CD9, anti-CD63, anti-CD81 antibodies, and negative control (PBS). Four blocking procedures were tested, and it was found that in combination with blocking procedures I and IV, the spotting buffers performed equally when analyzed fresh.

The reactivity of the spots was found to be preserved for up to 12 weeks when stored at RT and using blocking procedure IV in combination with trehalose in the spotting buffer. Similar preservation could be obtained using glycerol or sciSPOT D1 in the spotting buffers, but only if stored at 4 °C after the blocking procedure I.

Conclusively, it was found that immediate scanning of the MTPs after analysis was not critical if they were stored dried, in the dark, and at RT.

Within this study, we demonstrated the robustness of the antibody molecules and showed that EV phenotyping can be performed as a high throughput analysis in a 96-well format, which is a major step toward the implementation of EVs as biomarkers in a clinical setting.

## Data Availability

The data presented in this study are available on request from the corresponding author.

## Supplementary Materials

The following are available online at https://www.mdpi.com/article/10.3390/polym13142368/s1, Supplementary Materials S1: Materials tested in the pilot study.
