# Supplementary material for: Optimization of High-Throughput Multiplexed Phenotyping of Extracellular Vesicles Performed in 96-Well Microtiter Plates
Source: Polymers (Basel). 2021 Jul 19;13(14):2368. doi: 10.3390/polym13142368 (PMC8309600; doi:10.3390/polym13142368)
Supplement: Supplementary file 1 [file polymers-13-02368-s001.zip › polymers-1261604-supplementary.pdf]

Supplemental material S1

Materials tested in pilot studies. Data/ result not shown.

MTP plates

sciPLEX plate Type 1 (clear bottom)	Scienion AG (DE) # CPH-5511-10
3D Epoxy plate (clear bottom)	PolyAN GmbH (DE) # LW 160418
Corning standard, not treated (black bottom)	Corning Incorporated (MA, USA) # 3915
Corning standard, not treated (clear bottom)	Corning Incorporated (MA, USA) # 3631
Corning standard, high-binding (black bottom)	Corning Incorporated (MA, USA) # 3925
Corning standard, high-binding (clear bottom)	Corning Incorporated (MA, USA) # 3601
F96 Microwell™, non-treated (black bottom)	Thermo Fischer Scientific Inc. (MA, USA) #237108
Polysorp (black bottom)	Thermo Fischer Scientific Inc. (MA, USA) #437112
Maxisorp (black bottom)	Thermo Fischer Scientific Inc. (MA, USA) #437111
Optical-bottom plate, non-treated (clear bottom)	Thermo Fischer Scientific Inc. (MA, USA) #265301
Microfluor 1, medium binding (black bottom)	Thermo Fischer Scientific Inc. (MA, USA) #7605
Microfluor 2, high binding (black bottom)	Thermo Fischer Scientific Inc. (MA, USA) #7805
Universal binding (black bottom)	Thermo Fischer Scientific Inc. (MA, USA) #9502867

Spotting Buffers

5% Glycerol in PBS	
50 mM Trehalose in PBS	
sciSPOT D1	Scienion AG (DE) # CBP-5431-25
sciSPOT D11	Scienion AG (DE) # CBP-5435-25
sciSPOT D4	Scienion AG (DE) # CBP-5442-25
Carbonate buffer	50 mM Sodium Carbonate, pH 9.6

Blocking Buffers

TTN buffer	50 mM Tris, 1% Tween20®, 0.3 M NaCl, pH 7.5
1% Goat serum in PBS	Goat serum: Sigma Aldrich (MO, USA) # S26-LITER
1% BSA in PBS	BSA: Sigma-Aldrich (MO, USA) #7906
1x Casein Blocking Buffer	Sigma-Aldrich (MO, USA) # B6429
Ethanolamine	50 mM Ethanolamine, 0.1% SDS, 100 mM Tris, pH 9.0
Ethanolamine / 1x Casein Blocking Buffer	5x Ethanolamine concentrate diluted in 1x Casein Blocking Buffer
2% Tween20® in PBS	
sciBLOCK protein D1	Scienion AG (DE) # CBP-5433-500

Incubation and washing buffers

0.2 % Tween20® in PBS	
0.5% BSA + 0.1% Tween20® in PBS	
½x Casein Blocking Buffer + 0.1% Tween20® in PBS	
sciWASH protein D1	Scienion AG (DE) # CBP-5432-500
sciBIND protein D1	Scienion AG (DE) # CBP-5434-1.6
